# Ultrastructural localization of extracellular matrix proteins of the lymph node cortex: evidence supporting the reticular network as a pathway for lymphocyte migration

**DOI:** 10.1186/1471-2172-11-42

**Published:** 2010-08-17

**Authors:** Gregg P Sobocinski, Katherine Toy, Walter F Bobrowski, Stephen Shaw, Arthur O Anderson, Eric P Kaldjian

**Affiliations:** 1 MCDB Dept, University of Michigan, Ann Arbor, MI, USA; 2 Pathology Dept, University of Michigan, Ann Arbor, MI, USA; 3 Pfizer Global R&D, Groton, CT, USA; 4 NCI, Bethesda, MD, USA; 5 USAMRIID, Frederick, MD, USA; 6 Hearing Health Science, Ann Arbor, MI, USA

## Abstract

**Background:**

The lymph node (LN) is a crossroads of blood and lymphatic vessels allowing circulating lymphocytes to efficiently recognize foreign molecules displayed on antigen presenting cells. Increasing evidence indicates that after crossing high endothelial venules, lymphocytes migrate within the node along the reticular network (RN), a scaffold of fibers enwrapped by fibroblastic reticular cells (FRC). Light microscopy has shown that the RN contains specific extracellular matrix (ECM) proteins, which are putative molecular "footholds" for migration, and are known ligands for lymphocyte integrin adhesion receptors.

**Results:**

To investigate whether ECM proteins of the RN are present on the outer surface of the FRC and are thus accessible to migrating lymphocytes, ultrastructural immunohistochemical staining of cynomolgus monkey LN was performed using antibodies to human ECM proteins that were successfully employed at the light microscopic level. The fibrillar collagens I and III were observed primarily within the reticular network fibers themselves. In contrast, the matrix proteins laminin, fibronectin, collagen IV, and tenascin were observed within the reticular fibers and also on the outer membrane surface of the FRC.

**Conclusions:**

These findings suggest a molecular basis for how the RN functions as a pathway for lymphocyte migration within the lymph node.

## Background

The lymph node is a highly specialized organ in which circulating lymphocytes encounter processed foreign substances that are displayed on antigen presenting cells (APC). Blood-borne lymphocytes enter the node by transmigrating across high endothelial venules (HEV) [1-3]. From there, T and B lymphocytes travel to their specific compartments within the node [4,5]. The reticular network (RN) is a highly branched scaffold of inter-connecting fibers each of which is enwrapped by thin cytoplasmic processes of fibroblastic reticular cells [6,7]. It has been postulated that lymphocytes and APC use the RN as the route for trafficking within the node [4,5,8-10]. Recently, Bajénoff and Miller separately demonstrated the use of the reticular network as a path for lymphocyte trafficking using in vivo video imaging techniques [11,12,8].

Immunohistochemical staining at the light microscopic level has demonstrated that the RN contains a variety of extracelluar matrix (ECM) proteins, many of which are known ligands for integrin cell surface adhesion receptors [7]. Although these proteins could theoretically provide molecular "footholds" for cell trafficking along the RN [4,13], optical resolution does not allow the precise localization of ECM within the RN. It remains possible that these ECM proteins are present only within the fiber core - which is most of the volume of the RN - and not on the outer membrane surface of the enwrapping FRC. If so, the ECM proteins will not be available to migrating cells as stepping stones for movement, and would be unlikely to contribute to trafficking. We therefore undertook an ultrastructural immunohistochemical study in cynomolgus monkey lymph nodes to test the hypothesis that ECM proteins are accessible to migrating cells.

## Methods

### Care and Use of Laboratory Animals

All work was conducted in accordance with the current guidelines for animal welfare (Guide for the Care and Use of Laboratory Animals, 1996, Animal Welfare Act, 1996, as amended in 1970, 1976 and 1985, 9 CFR Parts 1, 2, 3).

### Light Microscopy

Lymph node slices from an adult female cynomolgus monkey were immersion-fixed in 10% neutral buffered formalin for 24 hours then routinely processed and embedded in paraffin. Paraffin sections were cut at 3 microns and air dried overnight. Heat-induced epitope retrieval, where indicated, was performed in a pressure cooker set at 5 lbs of pressure and 109°C while immersed in either EDTA pH 8.0 or citrate buffer pH 6.0 (Biocare Medical). Slides were cooled for 10 minutes, rinsed, and then stained and developed with diaminobenzidine using the automated NexesTM IHC Staining System (Ventana Medical Systems) using the manufacturer's reagents and protocols. Protease treatment was employed where indicated (Protease 2, Ventana). Primary antibody incubation time was 32 minutes. (See Table 1.)

**Table 1:** Histochemical (L.M.) Antibody Information.

Primary Antibody	Type, Species, IgG-type (Clone)	Dilution	Slide Pretreatment	Source	Secondary antibody	Dilution	Source
von Willebrand Factor	Polyclonal, Rabbit anti-Human IgG	1:2000	P2 = 16 min	Dako, A 0082	Universal link	Prediluted	Ventana Medical Systems

Collagen I	Polyclonal, Rabbit anti-Human IgG	1:100	(HIER) EDTA 5 min & P2 = 16 min	Biogenesis, 2150-0020	Universal link	Prediluted	Ventana Medical Systems

Collagen III	Polyclonal, Rabbit anti-Human IgG	1:100	(HIER) EDTA 5 min & P2 = 16 min	Biogenesis, 2150-0100	Universal link	Prediluted	Ventana Medical Systems

Laminin	Polyclonal, Rabbit anti-Mouse IgG	1:25	P2 = 32 min	Cappel, 10765	Universal link	Prediluted	Ventana Medical Systems

Fibronectin	Monoclonal, Mouse anti-Human IgG1 (Clone 568)	1:100	P2 = 32 min	Novo Castra, NCL-FIB	Universal link	Prediluted	Ventana Medical Systems

Collagen IV	Monoclonal, Mouse anti-Human IgG1 (Clone CIV 22(1))	1:100	P2 = 16 min	Dako, M0785	Universal link	Prediluted	Ventana Medical Systems

### Electron Microscopy

Lymph node pieces from an adult female cynomolgus monkey was immersed and minced in 1.25% glutaraldehyde and 2% paraformaldehyde fixative, fixed for 2-3 hours, rinsed, and stored in 0.1 M sodium phosphate buffer. The tissues were then dehydrated and embedded in LR White acrylic resin (Polysciences Inc.), and cold-cured under 365 nm UV light using a Pelco^® ^UVC2 Cryo Chamber (Ted Pella Inc). Ultrathin tissue sections were collected on nickel grids, and stained using the following steps: (1) 10 minute etch with 10% H_2_O_2_; (2) 10 minute pre-block with 1% BSA in PBS pH 7.4; (3) primary antibody incubation overnight at 4°C in PBS/BSA; (4) 2 hour secondary antibody incubation at room temperature in PBS/BSA; (5) 5 minute fixation in cacodylate-buffered 2.5% glutaraldehyde pH 7.2; (6) two, 5 minute silver enhancement incubations utilizing an IntenSE™M Silver Enhancement Kit (Amersham, RPN 491); (7) uranyl acetate and lead citrate counter stains of 8 minutes each. Rinses were performed between steps where appropriate. Primary antibodies and secondary 20 nm gold-conjugated anti-IgG antibodies were used at the dilutions listed in Table 2.

**Table 2:** Immunogold (E.M.) Antibody Information.

Primary Antibody	Type, Species, IgG-type (Clone)	Dilution	Source	Secondary antibody	Dilution	Source
von Willebrand Factor	Polyclonal, Rabbit anti-Human IgG	1:200	Dako, A 0082	Goat anti-Rabbit	1:100	BBI International, EM.GAR20

Collagen I	Polyclonal, Rabbit anti-Human IgG	1:100	Biogenesis, 2150-0020	Goat anti-Rabbit	1:200	BBI International, EM.GAR20

Collagen III	Polyclonal, Rabbit anti-Human IgG	1:200	Biogenesis, 2150-0100	Goat anti-Rabbit	1:100	BBI International, EM.GAR20

Laminin	Polyclonal, Rabbit anti-Mouse IgG	1:200	Cappel, 10765	Goat anti-Rabbit	1:50	BBI International, EM.GAR20

Fibronectin	Monoclonal, Mouse anti-Human IgG1 (Clone 568)	1:200	Novo Castra, NCL-FIB	Goat anti-Mouse	1:100	BBI International, EM.GAF20

Collagen IV	Monoclonal, Mouse anti-Human IgG1 (Clone CIV 22(1))	1:100	Dako, M0785	Goat anti-Mouse	1:50	BBI International, EM.GAF20

Tenascin-C	Monoclonal, Mouse anti-Human IgG1 (Clone TN2)	1:50	Dako, M0636	Goat anti-Mouse	1:100	BBI International, EM.GAF20

### Quantification

**Figure 1:**
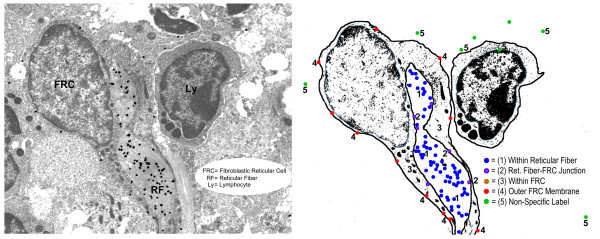
**Immunogold particle scoring method**.

Stained grids were viewed on a Philips CM 100 BIOTWIN transmission electron microscope. Images were captured using a digital camera (AMT Advantage CCD Camera System). For each stain, five separate fields (original magnification = 2,850×, area = approximately 65 square micrometers) were selected that included representative reticular fibers and associated fibroblastic reticular cells. These images were scored independently by two investigators (GPS, EPK) by counting silver-enhanced gold particles and categorizing according to location as described in Figure 1. A mean score was calculated. Variability in counts between investigators was within 3-5%.

In some cases, the area of evaluation contained a reticular fiber surrounded by a FRC, a part of which bordered a second adjacent fiber directly (which was in turn enwrapped by another FRC). These areas were understood to represent fiber branching points. Particles present on this membrane-fiber interface were considered part of the "outer reticular fiber" region rather than the "outer membrane" of FRC, since ECM here would not be accessible to migrating lymphocytes.

## Results

### Light microscopy immunohistochemistry

**Figure 2:**
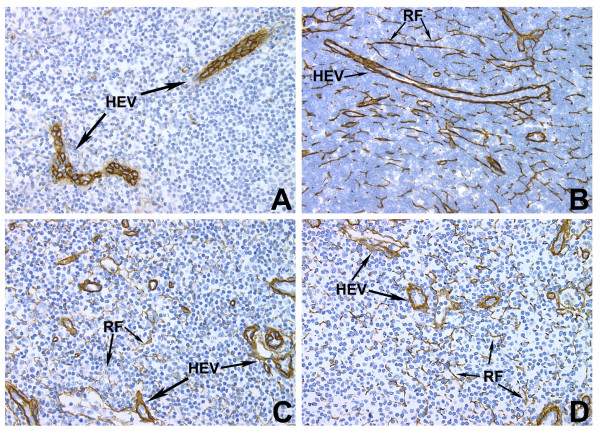
**Light microscopy immunohistochemical stains**. von Willebrand Factor stains vessels only (A). Collagen I (B), Collagen IV (C), and Fibronectin (D) all stain heavily within the reticular fiber network. (All images are same magnification).

Staining of monkey lymph node using anti-human ECM antibodies revealed a pattern similar to that observed in human lymph node [7,10,14]. Figure 2 shows representative images of collagen I and IV and fibronectin stains. Staining is observed along the reticular fibers and around blood vessels, where the fibers form sheaths that join the endothelial basement membrane, but is absent in endothelial cells. In contrast, von Willibrand Factor (vWF), an endothelial marker, is present in the endothelial cells only, and not the fibers. It is apparent that the limits of optical resolution do not allow determination of whether the ECM proteins are present on the outer membrane of the FRCs, within the reticular cells themselves, or within the fibers. (See Figure 2)

### Ultrastructural immunohistochemistry

**Figure 3:**
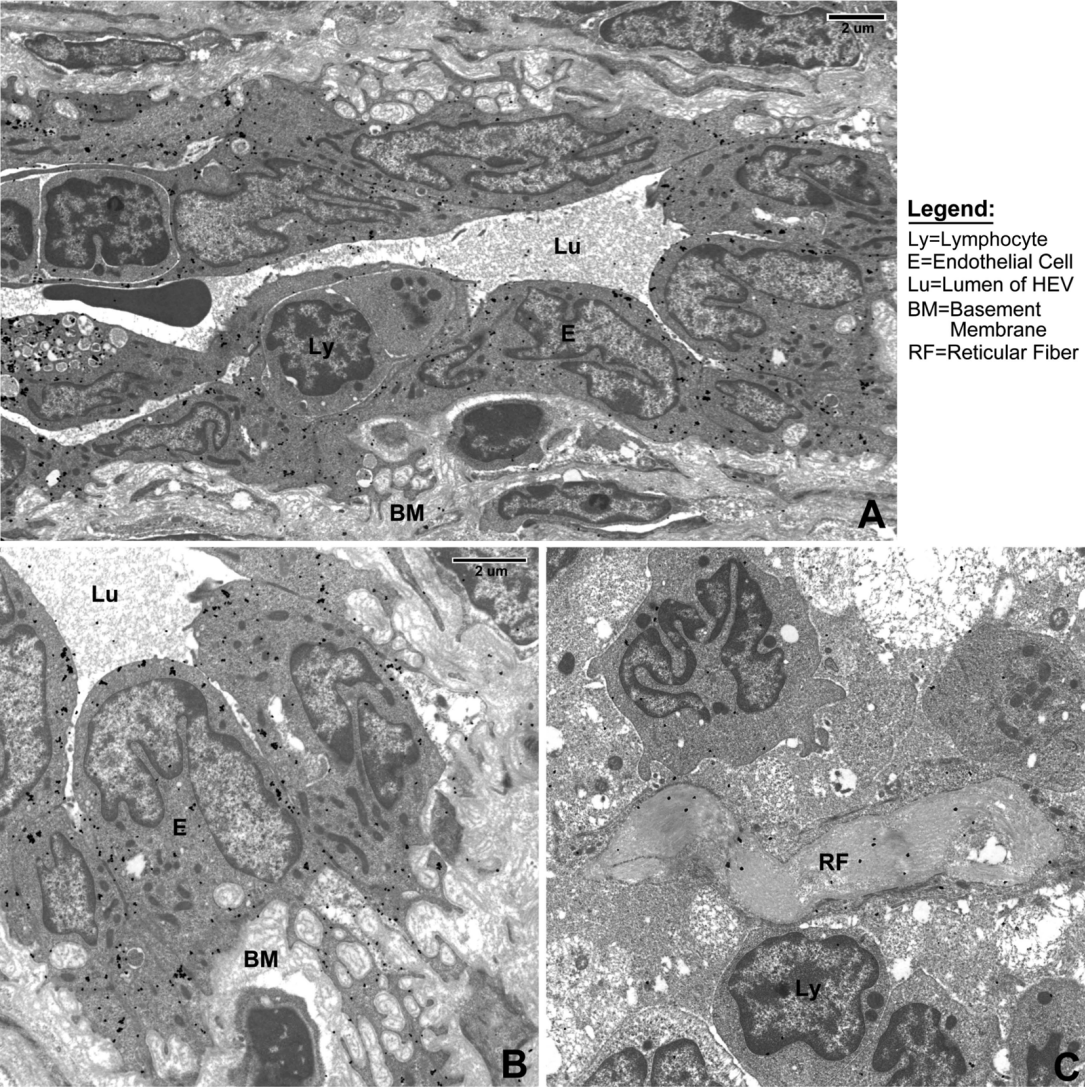
**von Willebrand Factor immunoelectron microscopic staining**. vWF is detected in the endothelial cells (A+B) of the HEV. Note lack of staining in the lymphocytes and the basement membrane matrix. Labeling in the reticular fiber (C) for von Willebrand Factor is non-specific. (Same magnification for images B and C).

**Figure 4:**
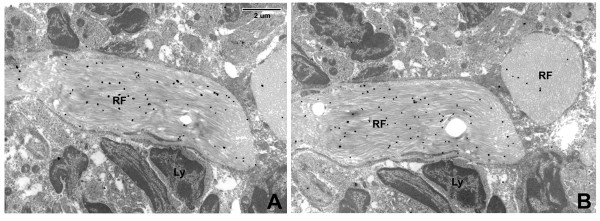
**Fibrillar collagen immunoelectron microscopy**. Collagen I (A) and Collagen III (B) are both easily detected within the reticular fiber of these serial sections. Label particles are more numerous in longitudinal section of reticular fiber (B, left RF) than in cross-section (B, right RF) (see discussion). (Same magnification for images A and B).

To technically evaluate the post-embedding immunohistochemical method, we stained monkey LN with antibody directed against von Willebrand factor (vWF). Silver-enhanced immunogold particles marked endothelial cells of the HEV present in the lymph node cortex. There was virtually no label observed in the subjacent endothelial basement membrane and background staining was minimal. Since staining of reticular fibers was equivalent to background levels throughout the section, we used the vWF stain as a negative control for non-specific staining in the quantitative assessment of ECM proteins (see Figure 3B and below). Since fibrils of collagens I and III are known to be present within the reticular fibers [7,10,15], we used stains for these collagens as positive controls. Label for both collagens was observed on the fibrils seen within the fiber, but not in the FRC or the surrounding tissues. It was noted that cross-sections of reticular fibers labeled less densely than did longitudinal sections (Figure 4B).

**Figure 5:**
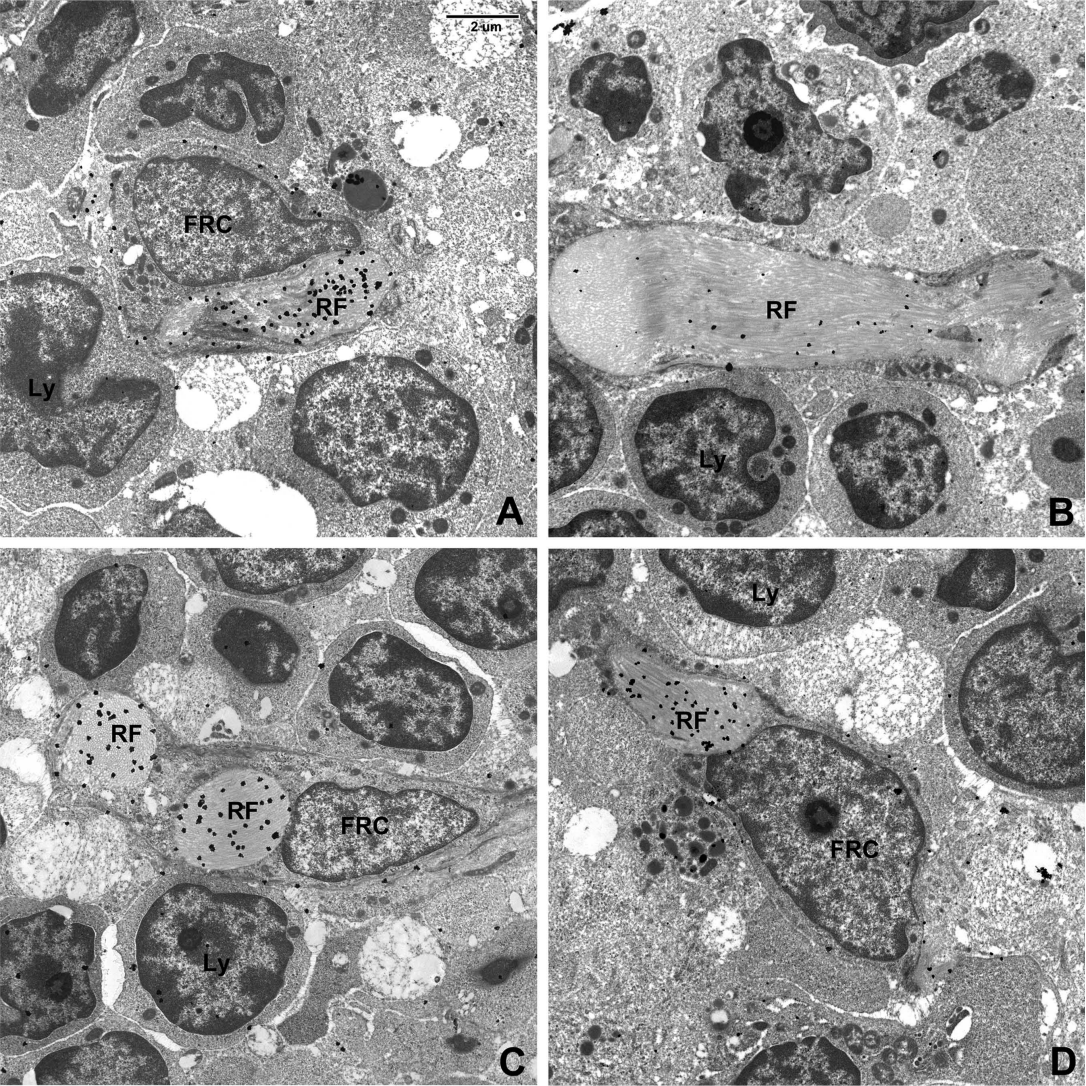
**Basement membrane extracellular matrix protein immunoelectron microscopy**. Collagen IV (A), Laminin (B), Tenascin (C), and Fibronectin (D) are all detected within the reticular fiber. In addition, the gold label is also present along FRC outer membranes. (All images are same magnification.)

**Figure 6:**
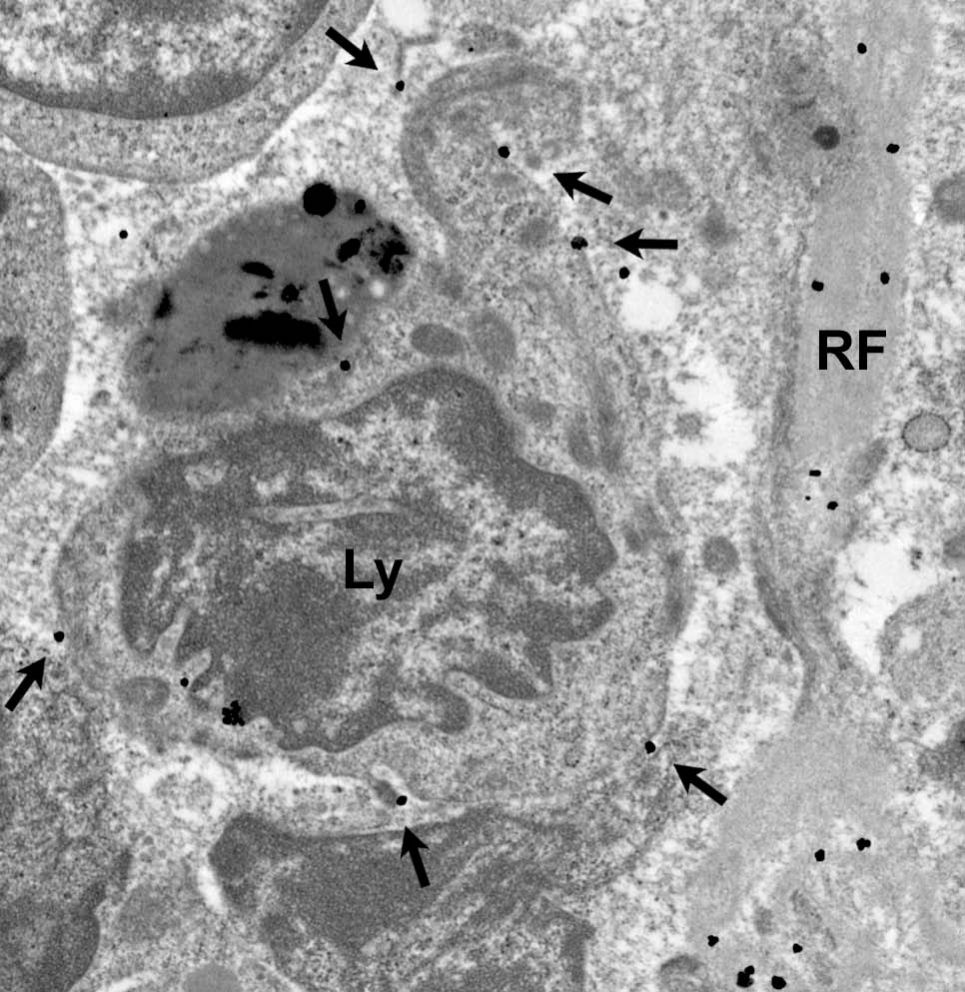
**Fibronectin on lymphocyte surface**. Several gold particles were observed along the cytoplasmic membrane of a lymphocyte.

Collagen IV, laminin, tenascin, and fibronectin are basement membrane-associated ECM proteins (hereafter referred to simply as "basement membrane proteins"). Ultrastructural immunostaining revealed that these proteins are found within the reticular fiber, but not in the FRC cytoplasm (Figure 5). Unlike collagens I and III, however, the basement membrane proteins were also detected on the outer membrane of the FRC. Representative stains for tenascin, collagen IV, and fibronectin (Figure 5 A, C, D) demonstrate electron-dense particles along the outer membrane of a FRC that enwraps a reticular fiber. There were rare examples of fibronectin staining along the outer membrane of a lymphocyte (Figure 6).

### Quantitative localization of staining

**Figure 7:**
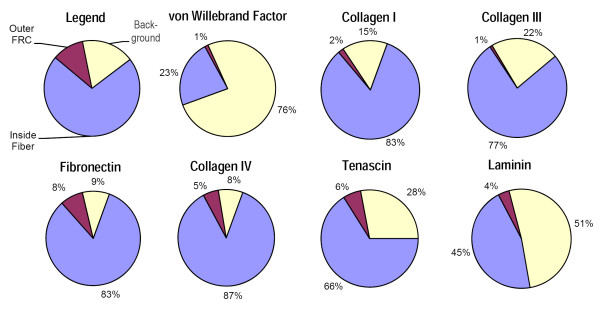
**Pie Chart Quantitation Summary**. The negative control vWF antibody primarily labels background structures, whereas the structural proteins collagen I and III are present primarily within the reticular fiber (RF); all three show minimal non-specific label on the outer FRC at ~1%. Fibronectin, collagen IV, and tenascin are distinctly present along the outer FRC and within the RF, with low background staining. Although the background level of laminin is considerably higher, it is nonetheless present along the outer FRC and within the RF at considerably higher amounts than the negative control vWF label.

In order to provide a quantitative correlate to our qualitative observations, we developed a scheme to score the silver grains on five representative images for each antibody stain. Grains were classified according to their location and then tallied. Figure 1 shows an image with a corresponding diagram that summarizes the scheme. The following micro-anatomic categories were used: (1) within reticular fiber, (2) at reticular fiber-FRC border, (3) within FRC cytoplasm, (4) at the FRC outer surface, and (5) non-specific background. Table 3 shows the mean results of scoring of the images by two investigators. After initial scoring, categories (1) and (2) displayed no independent discriminatory value, and so were combined to yield a score of all grains associated with the reticular fiber. Since there was no appreciable staining of FRC cytoplasm by any of the antibodies, grains in category (3) were considered non-specific background and combined with category (5). Using the resulting three categories, location percentages were calculated for each stain and pie charts were created to visualize the differences (Figure 7).

**Table 3:** Data-gold particles counted per area.

	Fibril Associated	FRC-Fiber Border	Total Within Fiber	FRC Cytoplasm	FRC Outer Membrane	Background	Background Plus FRC Cytoplasm	Total Grains Counted	Percent Within Fiber	Percent on FRC Outer Membrane	Percent Background ***
**vWF**	39	19	58	10	3	186	196	257	23	1	76
**Collagen I**	168	17	185	9	4	24	33	222	83	2	15
**Collagen III**	169	20	189	8	2	48	55	246	77	1	22
**Collagen IV**	449	144	592	5	36	51	56	684	87	5	8
**Laminin**	69	11	80	6	7	85	91	178	45	4	51
**Tenascin**	257	99	356	13	32	138	151	538	66	6	28
**Fibronectin**	275	84	359	4	34	37**	40	433	83	8	9

** 7 grains found on lymphocyte membrane in one fibronectin plate (See Figure 6.)

*** Immunogold granules found within the FRC (Figure 1, area 3) were added to the background tally (Figure 1, area 5) for determining the final statistics, since lack of significant FRC label implies merely background presence.

As explained previously, vWF was used as the negative control for reticular network staining. 76% of the (non-HEV) vWF label was in the background category, 23% within the reticular fiber, and 1% was associated with the FRC outer membrane. For collagens I and III the pattern of staining was reversed, with approximately 80% of the label within the fiber and most of the remainder in background; FRC outer membrane staining was 1-2%, comparable to vWF. Fibronectin and collagen IV stains yielded more than 80% of the label within the fiber, with background less than 10%. Tenascin scored 66% within the fiber, and had background of 28%. These three ECM stains had FRC outer membrane scores that ranged between 5 and 8%, which were considerably increased from the 1% level of vWF. Staining for laminin was weakest of all the stains. Although only 45% of the laminin label was within the fiber, this was still twice the amount present in the vWF control, indicating positive staining. In addition, 4% of the grains were counted on the FRC outer membrane.

## Discussion

It has been logically argued that both lymphocytes and antigen presenting cells travel along the RN to reach their respective compartments, since within the node cells "crawl but they can't swim" and thus need a road on which to travel [16,7]. Recently, video imaging of fluorescently labeled cells in a murine in vivo system has demonstrated real-time interaction between lymphocytes and the cells of the RN as they traffic through the node [11,8]. Fibronectin [17,18], collagen IV, tenascin, and laminin, are basement membrane (BM) ECM components present within the RN [10,19-22] that are known ligands for integrin adhesion receptors, influencing cell taxis *in vitro*. Consequently, it has been postulated that such ECM proteins associated with the RN participate in cell migration across HEV and through the LN [23-26,16,7,5,9,10]. The known ultrastructure of the RN does not require this to be the case, however, since the matrix components could be confined to the inner region of cable-like collagen fibrils, enwrapped by the thin FRC covering that separates it from the cavernous spaces of the nodal cortex [7]. Given the thinness of the wrapping, the precise location of the matrix proteins is optically irresolvable. By immunoelectron microscopy, however, we were able to localize them to specific regions of the RN.

Like the fibrillar collagens I and III that form the skeletal structure of the RN, the BM ECM proteins were observed within the reticular fiber. Previous studies have shown that the reticular network is a conduit for solutes to pass quickly from the afferent lymphatics entering the sub-capsular sinus to the HEV [27,28,5]. It is possible that these BM ECM components within the reticular fiber may participate in this solute transport. But unlike the fibrillar collagens, the BM ECM proteins were also detected on the outer membrane of the FRC - where they are accessible to migrating cells - at a level that was several times greater than background (see Figure 4; Tables 3 and Figure 7). Our observations thus confirm that the RN contains a complex mix of basement membrane-type proteins, and that they are present both within the fibers and on the outer FRC cell surface, where they can function as adhesion ligands for cell migration. This information provides experimental evidence for what had been hypothesized as the basis for cell crawling that is consistent with understood leukocyte motility. Moreover, this has been demonstrated in non-human primate lymph node, which closely resembles its human counterpart.

We observed the occasional absence of FRC cellular processes completely enwrapping the reticular fibers. It has been previously proposed that such gaps in the covering of the reticular fibers may allow direct access to reticular fiber ECM for migrating cells and thus provide the substrate for cell crawling. Ohtani [29] observed "fenestrations" in the FRC of rabbit Peyer's patches, most notably in flat cell processes. Ushiki [30] described incomplete wrapping of fibers in the rat node that was prominent in the medulla but rare in the deep cortex. Our studies were performed on non-human primate lymph nodes that closely resemble human lymph nodes, and investigated the T cell cortex, equivalent to the "deep cortex" of the rat. In this region the RN is made mostly of rope-like cords, rather than sheets, of fibers, and the FRC cell processes covering the fibers are not flat, but tubular. Our findings thus appear consistent with reports that exposed fibers in this region are not commonly found.

It is possible that we have underestimated the amount of membrane-associated ECM relative to fiber-associated ECM. The FRC membrane appears as a one-dimensional, linear structure in sections prepared for transmission electron microscopy. In contrast, the fiber typically appears as a broad, two-dimensional plane. Thus, there is considerably less FRC membrane surface area than fiber surface area in the image fields evaluated. Our method tallied the total number of grains per image field, but did not calculate the density (the number of grains per unit area of membrane or of fiber) of the label within each region category, since defining the thickness of the membrane proved to be too subjective. The density, therefore, of the membrane-associated staining may actually be much higher relative to the density of the fiber-associated staining than the absolute number counts suggest.

Two other notable findings of the label particle distribution were observed in our study. Rarely, as shown in the fibronectin stain in Figure 6, silver grains were observed ringing the outer membrane of individual lymphocytes. It is possible that this represents synthesis by the lymphocyte, which then expresses the fibronectin at its surface; indeed, this has been described by Wagner and colleagues [31]. Alternatively, it may be that as the lymphocyte has traversed through and along the ECM of the node, fibronectin has "stuck" to integrin receptors on the cell surface. In addition, when staining for collagen I and III, we noted increased density of silver grains in longitudinal views of reticular fibers versus cross-sectional views (Figure 4-B). This may imply that the binding epitope is on the outer surface of the collagen triple helix, or somehow simply more accessible from the "side".

It is tempting to speculate on how the expression of adhesion ligands by FRC may guide the migration of cells to specific locations. Since the FRC wrap the reticular fibers, they are directly exposed to the solutes that pass through the FRC conduit from the sub-capsular sinus to the HEV. Thus, they are well-positioned to respond to factors that should logically prompt an increase in immune trafficking, and could increase their surface adhesion ligand density to encourage more efficient migration within the node and a corresponding higher rate of contact with APC [11].

These findings, if substantiated, may have implications for the understanding of immunological diseases [24]. Since direct contact between APC and lymphocyte essential to the generation of immune response, blocking such contact could suppress the abnormal immune activation in autoimmunity. One way to disallow contact is to keep the cells from being able to meet. If travel within the node is in fact regulated by BM ECM proteins on the outer surface of the RN, blocking ligand-adhesion receptor binding with inhibitors could keep the cells from crawling to their correct compartments, and so dampen immune response. Conversely, in disorders of ineffective immunity, weakened immune response may exist from an endogenous inhibition of migration by unknown factors, or by too little BM ECM than required for adequate intra-nodal trafficking. Finally, it is conceivable that metastatic cancer cells may use similar mechanisms for traveling within the node. Thus, modulating the migrating cell-reticular network interaction may be a rational, albeit challenging, therapeutic approach.

## Conclusions

We have demonstrated that ECM proteins known to be ligands of immune cell adhesion receptors are expressed on the outer surface of FRC within the cortex of non-human primate lymph nodes. These findings provide a molecular basis for how the RN may function as a pathway for cell migration within the lymph node.

## List of abbreviations

LN: Lymph Node; RN: Reticular Network; FRC: Fibroblastic Reticular Cell; ECM: Extracellular Matrix; APC: Antigen Presenting Cells; HEV: High Endothelial Venules; vWF: von Willebrand factor; BM: Basement Membranes.

## Authors' contributions

GS- Electron microscope sample collection, immunocytochemistry, data collection and analysis. Created manuscript, plates, and figures. KT- Provided immunohistochemical data and interpretation. WB- Electron microscopy and immunohistochemistry consultation. Manuscript assembly and formatting assistance. SS- Collaboration toward the theory behind the research and interpretation of results. AA- Collaboration toward the theory behind the research and interpretation of results. EK- Designed the experiment. Data collection, interpretation, assisted manuscript writing, and editing. All authors read and approved the final manuscript.

## Authors' Information

Gregg P. Sobocinski BSc. CEMT(MSA), QIHC(ASCP). Microscope Imaging Specialist, University of Michigan, Molecular, Cellular, & Developmental Biology Department, Ann Arbor, MI, USA.

Katherine Toy. HT(ASCP) QIHC(ASCP). Research Laboratory Specialist, University of Michigan, Pathology Department.(Internal Medicine/Oncology), Ann Arbor, MI, USA.

Walter F. Bobrowski BSc., CEMT(MSA). Senior Scientist, Pfizer Global R&D, Groton, CT, USA.

Stephen Shaw PhD. Head of Human Immunology Section, Experimental Immunology Branch.  NCI, Bethesda, MD, USA.

Arthur O. Anderson PhD Director, Office of Human Use and Ethics, Research Integrity Officer US Army Medical Research Institute of Infectious Diseases, Frederick, MD, USA.

Eric P. Kaldjian MD, Medical Director, Hearing Health Science, Ann Arbor, MI, USA.
